# Identification and impact of stable prognostic biochemical markers for cold-induced sweetening resistance on selection efficiency in potato (*Solanum tuberosum* L.) breeding programs

**DOI:** 10.1371/journal.pone.0225411

**Published:** 2019-12-31

**Authors:** Sanjay K. Gupta, James Crants

**Affiliations:** University of Minnesota, Department of Soil, Water and Climate, St. Paul, MN, United States of America; United Arab Emirates University, UNITED ARAB EMIRATES

## Abstract

Biochemical markers for cold-induced sweetening (CIS) resistance were tested for their stability over years and their use in selection of parents for crossing to achieve high selection efficiency in potato breeding programs.

Two regulatory enzymes directly associated with reducing sugar (RS) accumulation during potato tubers cold storage were tested as a predictor for CIS resistance. These enzymes were studied in 33 potato clones from various breeding programs over four years. Clones with the presence of A-II isozymes of UDP-glucose pyrophosphorylase (UGPase) and low activity of vacuolar acid invertase (VAcInv) enzyme had increased resistance to cold-induced sweetening (CIS). Depending on the levels of these enzymes, clones were divided into class A, class B and class C. Clones categorized as class A had average RS of 0.73 mg per g FW after six months at 5.5°C storage. Class B and C had average RS of 1.15 and 3.80 mg per g FW respectively. The enzyme activity was closely associated with RS accumulation over long-term cold storage.

The biochemical markers were found to be stable over the years. Repeated-measure analysis showed 75% chance of maintaining class from one year to the next and a 25% chance of switching, No clone switched between class A and class C, even across all four years. Application of these biochemical markers can identify clones with CIS resistance early in the selection process. Biochemical markers were used to select parents for crossing and six families were established. Results showed that with both parents from class A, 95% of their offspring had desirable glucose levels and chip color, which dropped to 52% when one parent was from class A and other from class B. These results suggest that two regulatory enzymes, i.e., UGPase and VAcInv, can be used as stable prognostic biochemical markers for CIS resistance for precise parent selection resulting in progenies with significantly higher percentage of clones with acceptable processing quality.

## Introduction

In the USA about 61% of total potato production is used for processing into French fries, chips or granules. Potatoes for processing are commonly stored at 8 to 12°C cold storage to prevent accumulation of RS [1,2]. However, storage of potatoes at low temperatures, 2 to 4°C, has several advantages including lower disease pressure, less shrinkage and water loss, reduced chemical use and longer storage and marketing window. These benefits are compromised at 2 to 4°C by a physiological process commonly known as cold-induced-sweetening (CIS), whereby starch is degraded to produce excessive amount of RS, i.e., glucose (glc) and fructose (fru). In USA, 15% of the processing potatoes are lost annually due to high levels of RS accumulation [3].

High levels of RS are the most important factor that adversely affect French fry and chip quality. Potatoes with high levels of RS produce unacceptable products through the non-enzymatic Maillard reaction during processing. Conventionally, processing quality during storage is monitored through French fry color and RS level tests at regular intervals. These methods have been used routinely in the processing industry as they are simple but time consuming, and only provides a tuber quality assessment at that given time. In addition, French fry color and sugar tests cannot predict the tuber quality change during cold storage.

In potato tubers, amylolytic enzymes in the amyloplasts convert starch to Glc-6-P, which can then be diverted for energy production through respiration or hexogenesis pathway to produce RS, depending on the isozymes of UGPase enzyme [4]. The rate of starch breakdown is generally not limiting in potato tubers. An increase in starch phosphorylase (EC 2.4.1.1) activity during cold storage acts as a triggering event for the accumulation of RS in potato tubers [5–7]. The hexogenesis pathway involves four enzymes, namely UDP-glucose pyrophosphorylase (UGPase EC 2.7.7.9), sucrose 6-phosphate phosphatase (S-6-P EC 3.1.3.24), sucrose phosphate synthase (SPS EC 2.4.1.21), and vacuolar enzyme acid invertase (VAcInv EC 3.2.1.26). The hexogenesis pathway is described in review article by Sowokinos [8]. Of the four enzymes, two enzymes namely UGPase and VAcInv are considered key regulatory enzymes. They are responsible for excessive accumulation of RS during long term cold storage [9,10]. The first regulatory step in hexogenesis pathway is the conversion of Glc-1-P to the nucleotide sugar uridine diphosphate-glucose (UDP-glc), mediated by the enzyme UGPase [11]. High energy UDP-glc is subsequently gets converted to sucrose (suc) with the action of SPS and S6P enzymes.

A relationship between chip color and lower levels UGPase has been demonstrated [9,12]. CIS resistant potatoes are shown to have isozymes of UGPase commonly referred to as A-II protein or A-II isozymes of UGPase [12]. Activity of UGPase may be related to abiotic stresses [13]. For example, a change in UGPase enzyme activity in response to heat stress [14] and moisture stress has been reported [15].

The final step in hexogenesis pathway is catalyzed by the vacuolar enzyme VAcInv (EC 3.2.1.26). VAcInv catalyzes the irreversible hydrolyses of suc to RS glc and fru [16,17]. A correlation between VAcInv activity and the hexose:sucrose ratio has been reported in potato cultivars [10,18–20]. Potato cultivars with low levels of VAcInv activity produced light chip color [10,21]. Cold temperature has been reported to increase transcription [20] and total acid invertase enzyme activity [22].

The degree of increase in VAcInv enzyme activity depends on genotype, storage temperature and the level of acid invertase inhibitor protein present in the tuber. Xu et al. [23] reported low levels of RS in potato clones with low levels of VAcInv.

Potato clones with A-II isozymes of UGPase and low levels of VAcInv activity had high resistance to CIS [4,11].

The main objective for most of the chipping industry is to develop new potato cultivars with low potential to accumulate excessive RS during storage [24,25]. New cultivar development uses a traditional strategy of outcrossing and then screening progenies to identify improved lines. This strategy is labor intensive, time consuming, and expensive and often takes more than 15 years to develop a new cultivar with improved quality. The conventional breeding strategy employs phenotypic selections over several generations [26]. Generally, large breeding populations are created and then reduced over the years through clonal selection process. In most of the breeding programs, often more than 99 percent of breeding clones are discarded in first two years of selection.

Researchers have used molecular genetic markers to accelerate potato breeding efforts known as Marker Assisted Selection (MAS) [27–30]. MAS proved effective when used on G-2 populations compared to traditional screening [30]. Most of the markers for MAS were for disease resistance [31] and depend on genetic maps to identify markers linked to the phenotypic trait of interest [32]. Slater el al. used a combination of MAS, EBVs (Estimated Breeding Value) and conventional screening methods for two diseases potato cyst nematode (PCN and potato virus Y (PVY) to reduce the length of breeding cycles and the cost of breeding [31]. DNA markers for CIS have been developed with limited success [33] and their cost is prohibitive for screening large populations. Better methods of selection of suitable parents are needed to improve potato breeding efficiency.

The goals of this study were to test the stability of two key regulatory enzymes, i.e., UGPase and VAcInv, as predictive selection markers for parent selection to obtain high frequency of clones with improved CIS resistance and to screen a large number of early generation clones. The advantage of identifying parents whose progenies will have a much higher percentage of acceptable processing clones is that it reduces the costs significantly compared to current breeding programs where progenies have a much lower percentage of acceptable processing clones. These biochemical markers are lab based tests and can be used along with traditional fry color and reducing sugar test. Unlike traditional tests, these markers are predictive in nature and therefore, can be used once at harvest without any further tests during storage.

In this study, we (1) validated the stability of biochemical markers for CIS resistance and (2) used biochemical markers for parent selection to obtain high selection efficiency.

## Materials and methods

### Reagents

All chemicals, reagents, enzymes were purchased from Sigma Chemical Company, unless otherwise stated. Molecular weight standards, and protein assay dyes were purchased from BioRad Laboratories.

### Plant material

Thirty-three potato cultivars and clones from various potato breeding programs, ID, MN, ND, MI, NY, CO, and ME, were planted in May for four years (2006, 2007, 2008 and 2009) at Larimore, ND, in an Embden fine sandy loam soil with 3.67% organic matter and a pH of 7.3. Local commercial practices were followed for cultivation, fertilization, and disease control. Potatoes were harvested in September, and after two weeks of reconditioning in room temperature (13°C and 95% relative humidity), tubers were placed in 5.5°C storage at the USDA-ARS Potato Research Worksite, East Grand Forks, MN. Samples were collected from tubers for enzyme and sugar analysis at zero time analysis before being placed in cold storage. Storage temperature was decreased to 5.5°C for all the clones and cultivars.

In order to gain a better understanding of the biochemical markers use in parent selection for crossing, breeding clones used in the University of Minnesota Potato Breeding Program were sampled and subsequently divided into 3 main classes (A, B, or C) as described earlier [34]. Category A—best CIS resistance, B—intermediate CIS resistance, and C—low CIS resistance. Six families were established based on CIS resistance classes. Three families 142 (MN99380-1Y * MN02696), 148 (ND860-2 * MN99380-1Y), and 138 (MN02696 * Waneta) were each produced by crossing two class A lines. In contrast, families 161 (W6609-3 * Snowden), 126 (Atlantic * Waneta) and 127 (Atlantic * Lamoka) were produced by crossing class A lines with class B lines. Crosses among these parents were made in 2013. Mini tubers generated in the greenhouse were planted in the field using normal plant spacing and production practices. To increase tuber numbers, these families were planted in May 2014 at Gully, MN, in Strathcona fine sandy loam soil with 5.12% organic matter and a pH of 7.1. After harvesting in November 2014, tubers were reconditioned for three weeks at room temperature. Tuber samples were collected after reconditioning for specific gravity, chip color, RS and Suc evaluation at zero time. The rest of the tubers were stored at 5.5°C storage for evaluations during cold storage.

For enzymatic analysis, a cross-sectional slice that included periderm, cortex, and pith was cut and five grams of tissue was taken from each of three tubers, immediately frozen in liquid nitrogen, and ground under liquid nitrogen using a freezer mill Spex 6850 (SPEX SamplePrep, NJ, USA). The frozen powder was stored at -80°C freezer until further analysis. Three replications of each clone were analyzed at 6 months.

### Chip color and specific gravity measurements

At each sampling period, six tubers of each clone were washed, sliced and processed on a pilot-scale chip fryer at 180°C for 90 sec. Chip color determination was made visually on 20 chip samples using the Snack Food Association Color Standards Reference Chart for Potato Chips [12]. Color scores of 1 and 2 = acceptable, 3 = marginal, and 4 and 5 = unacceptable chip color.

The specific gravity of the tubers was calculated using the following formula: Specific Gravity (SG) = weight-in-air/(weight-in-air–weight-in-water) [25].

### Determination of tuber sugar concentration:

Total sugar concentration was determined as previously described [34]. A YSI model 2000 Industrial Analyzer (Yellow Springs Instruments Co., Inc., Yellow Springs, OH) was used to estimate Glc and Suc. The concentration of Glc and Suc was expressed in mg g^-1^ FW. Tuber total sugar was calculated using the formula: Total sugar = (glc concentration *2) + suc concentration.

### Enzyme extraction and desalting

Total enzyme extraction and VAcInv activity assays were conducted at 4°C as previously described [34]. The extraction buffer contained 50 mM HEPES (pH 7.5), 15 mM MgCl_2_, 2 mM EDTA, 2 mM dithiothreitol (DTT), 10% v/v glycerol and 2% PVPP, 2.0 μg mL^-1^ leupeptin, and 1 mM PMSF. Supernatant was desalted with 2 mL desalting column (ThermoFisher Scientific, NY, USA). Desalting columns were pre-equilibrated with desalting buffer containing 50 mM HEPES (pH 7.5), 15 mM MgCl_2_, 2 mM EDTA, 2 mM DTT, and 2.0 μg mL^-1^ leupeptin. Desalted extracts were stored at -80°C freezer until assay.

#### Vacuolar acid invertase (basal and total activities) assay

VAcInv enzyme activity in tubers was assayed according to previously a published protocol [34]. Basal VAcInv enzyme activity was assayed in the presence of its inhibitor protein.

For determination of total VAcInv activity, 500 μL of the desalted extract was vortexed at maximum speed for 20 min to effectively remove its inhibitor. Foamed aliquots were centrifuged for five min at 23000 g. The invertase inhibitor was removed with vigorous foaming [35].

In each basal or total VAcInv activity aliquot, glc liberated from hydrolysis of suc was quantified by adding 100 μL Sumner’s reagent [44 mM 3,5-dinitrosalicylic acid (DNS), 2 M sodium hydroxide and 940 mM potassium sodium tartrate] [36]. The reduction of DNS to 3-amino-5-nitrosalicylic acid by glc was measured at A550 nm using a microplate reader (BioteK Elx808, Vermont, USA). Quantitation of glc in each sample was based on glc standards and VAcInv activity was expressed as μmol glc formed h^-1^ in the presence or absence of its endogenous inhibitor.

### Protein determination

The dye-binding method [37] was used for the determination of protein concentration in the samples. Bovine gamma-globulin (BGG) was used as the protein standard.

### A-II isozymes of UGPase detection

The presence of A-II isozymes of UGPase was detected using enzyme activity staining on a native 7.5% polyacrylamide gel [38]. The gel was run according to the procedure of Laemmli [39]. Previously purified UGPase was used as the standard protein [9,40].

### Cold-sweetening resistance (CIS) class

Based on basal VAcInv enzyme activity and their resistance to CIS a subjective scale of 1 to 3 units of basal VAcInv activity [34] was developed and potato clones categorized as class A (<1, best CIS resistance), cass B (1–3, intermediate resistance) and class C (>3, CIS susceptible). Clones with and without A-II isozymes of UGPase were designated with ‘+’ or ‘-‘ sign (S1 Table).

### Statistical analysis

Data were analyzed with SAS 9.4m3^®^ software (copyright 2015, SAS Institute, Inc.). Repeated-measures analyses were performed of basal VAcInv activity, total VAcInv activity, and tuber glc and suc concentrations as functions of CIS class, year, and their interaction, with year as the repeated-measures variable, clone as the subject variable, and a compound-symmetrical covariance matrix structure. Marginal means for dependent variables were determined using the LSMEANS statement, and post-hoc pairwise comparisons (alpha = 0.05) were conducted using the DIFF option. Pairwise comparisons are only presented where the significance (P-value) of the corresponding effect in the model is less than 0.05.

The hypothesis that the rank orders of clones by CIS class and basal VAcInv activity rarely change across years was evaluated with Friedman analysis using the FREQ procedure.

## Results and discussion

The two biochemical markers were studied over four years to study the stability over the years and were tested in a potato breeding program to select promising parents for crossing and screening of progeny for CIS resistance. Results on biochemical markers are presented and discussed in terms of their association with reducing sugar accumulation, values as predictors of CIS resistance during cold storage and finally their use in precise parent selection for crossing.

### Basal and total acid invertase activity

Basal VAcInv enzyme activity, averaged across the 33 clones included in the four-year study, varied significantly among the four years (P = 0.0098), being higher in 2009 than in the three previous years. Because the repeated-measures analysis takes clone identity into account, this yearly variation in enzyme activity is attributable to environmental effects and is not an artifact of the particular clones included in 2009. No similar effect of year was observed in total VAcInv enzyme activity or tuber glc or suc concentrations.

The basal VAcInv enzyme activity averaged over three to four years was lowest in class A followed by class B and class C (Table 1). Class A clones and cultivars averaged 0.82 units of basal VAcInv enzyme activity. Among class A clones and cultivars, the lowest average basal VAcInv enzyme activity was recorded in clone ND5255-59. Over three years, the basal VAcInv enzyme activity remained below 1 unit (S1 Table).

**Table 1:** ANOVA and mean comparison of basal and total VAcInv enzyme activity over four years. Values within a column that have a lowercase letter in common and values within a row that have an uppercase letter in common are not significantly different in pairwise comparisons (α = 0.05). Pairwise comparisons were only made for effects that were statistically significant in the ANOVA model (α = 0.05).

Basal VAcInv activity	Year				
(units·mg protein^-1^·hr^-1^)					
CIS class	2006	2007	2008	2009	Average
					across years
A (12 lines)	0.85	0.62	0.82	1.00	0.82 c
B (13 lines)	1.43	1.55	1.49	1.74	1.55 b
C (8 lines)	4.12	3.88	4.66	5.81	4.62 a
Average across CIS classes	2.13 B	2.02 B	2.32 B	2.85 A	-
Effect of CIS class (P-value)	**<0.0001**				
Effect of year (P-value)	**0.0098**				
Effect of CIS class * year (P-value)	0.1395				
Total VAcInv activity	Year				
(units·mg protein^-1^·hr^-1^)					
CIS class	2006	2007	2008	2009	Average
					across years
A (12 lines)	5.70	3.75	4.44	5.38	4.82 c
B (13 lines)	15.38	9.83	12.09	8.06	11.34 b
C (8 lines)	22.87	21.47	17.58	17.58	19.87 a
Average across CIS classes	14.65	11.68	11.37	10.34	-
Effect of CIS class (P-value)	**<0.0001**				
Effect of year (P-value)	0.1299				
Effect of CIS class * year (P-value)	0.4522				

A higher basal VAcInv enzyme activity of 1 unit was recorded in clone ND5775-3. Atlantic and MSN191-2Y showed wide variation in basal VAcInv enzyme activity over years but their average was below 1 unit. Therefore, on average they were classified as class A but in one year were classified as class B. This variation in basal VAcInv enzyme activity over years could be attributed to their sensitivity to environmental stresses, including water and temperature stress. Such stresses could induce higher basal VAcInv enzyme activity [15] to produce more RS as a protectant. Zhu et al. [41] reported that silencing the VAcInv gene could be used to reduce the sugar-end defects in French fries. Clasen et al. [42] improved CIS resistance and processing quality in potatoes through targeted gene knockout. Liu et. al. [43,44] and Ou et al. [45] reported significantly improved color in antisense lines. High basal VAcInv activity after six months’ storage could also be due to physiological maturity of tubers at the time of harvesting. Studies have shown an increase in VAcInv activity in response to cold storage [20,22,46]. None of the cultivars and clones in class A had basal VAcInv enzyme activity of over 3 units (class C) in any of the four years. It is interesting that Ou et al. [45] reported significantly improved color in antisense lines when invertase activity was near 5 μmols glc h^-1^ g^-1^ FW. Using an average of 6 mg protein g^-1^ FW of tuber, this would equate to an SA of 0.83. Therefore, basal VAcInv enzyme activity of <1.0 is recommended for improved resistance to CIS.

Among class B clones, the average basal VAcInv enzyme activity was 1.55 units, ranging from 0.35 units in Lelah to 2.91 units in Snowden (S1 Table). Most of the cultivars and clones in class B remained in class B with few exceptions. Lelah, Sport860, Dakota Crisp, W2978-3, W2683-2Rus, and Clearwater Russet each showed basal VAcInv in class A once in three years. None of them were in class C. The difference in average basal VAcInv enzyme activity between classes was statistically significant (Table 1). For class C cultivars and clones, the average basal VAcInv enzyme activity was 4.62 units (Table 1). All the clones remained in class C over years, with one exception. Clone MN15620 had basal VAcInv enzyme activity of 2.16 (class B) once in four years. None of the class C clones had basal VAcInv activity of less than 1 in any year.

Basal VAcInv enzyme activity has been associated with RS in potato tubers under moisture stress [15,35,47]. However, total VAcInv activity (i.e., assayed after destroying the endogenous inhibitor) showed a better correlation with change in RS than basal VAcInv enzyme activity [18] possibly due to a varying amount of inhibitor protein present. The increase in VAcInv enzyme activity after destroying endogenous invertase inhibitor protein is a direct reflection of the amount of inhibitor protein. Variations in total VAcInv enzyme activity over years were observed. Class A showed an average total VAcInv activity of 4.82 units, followed by class B clones with an average of 11.34 units, and class C clones with an average of 19.87 units (Table 1). A parallel increase in inhibitor protein with VAcInv enzyme reflects co-expression of both proteins [44,45,48–50]. A potato cultivar or clone with high levels of invertase inhibitor protein at harvest could lose its CIS resistance over long term cold storage, depending on total VAcInv activity. One good example is the Premier Russet, which is known to have good initial CIS resistance, but towards the end of storage, it accumulates high levels of RS (unpublished results).

It is interesting to note that Waneta (1.58 units), Lamoka (2.05 units) and Lelah (2.6 units) consistently had the lowest total VAcInv activity over years (S2 Table). These three cultivars are known to maintain their CIS resistance during the entire storage season. They showed consistently low levels of glc over 4 years (S3 Table). Class C clones and cultivars showed high levels of total VAcInv enzyme activity (S2 Table) and high glc concentrations (S3 Table).

### Relationship between enzyme activity and reducing sugar

**Fig 1:**
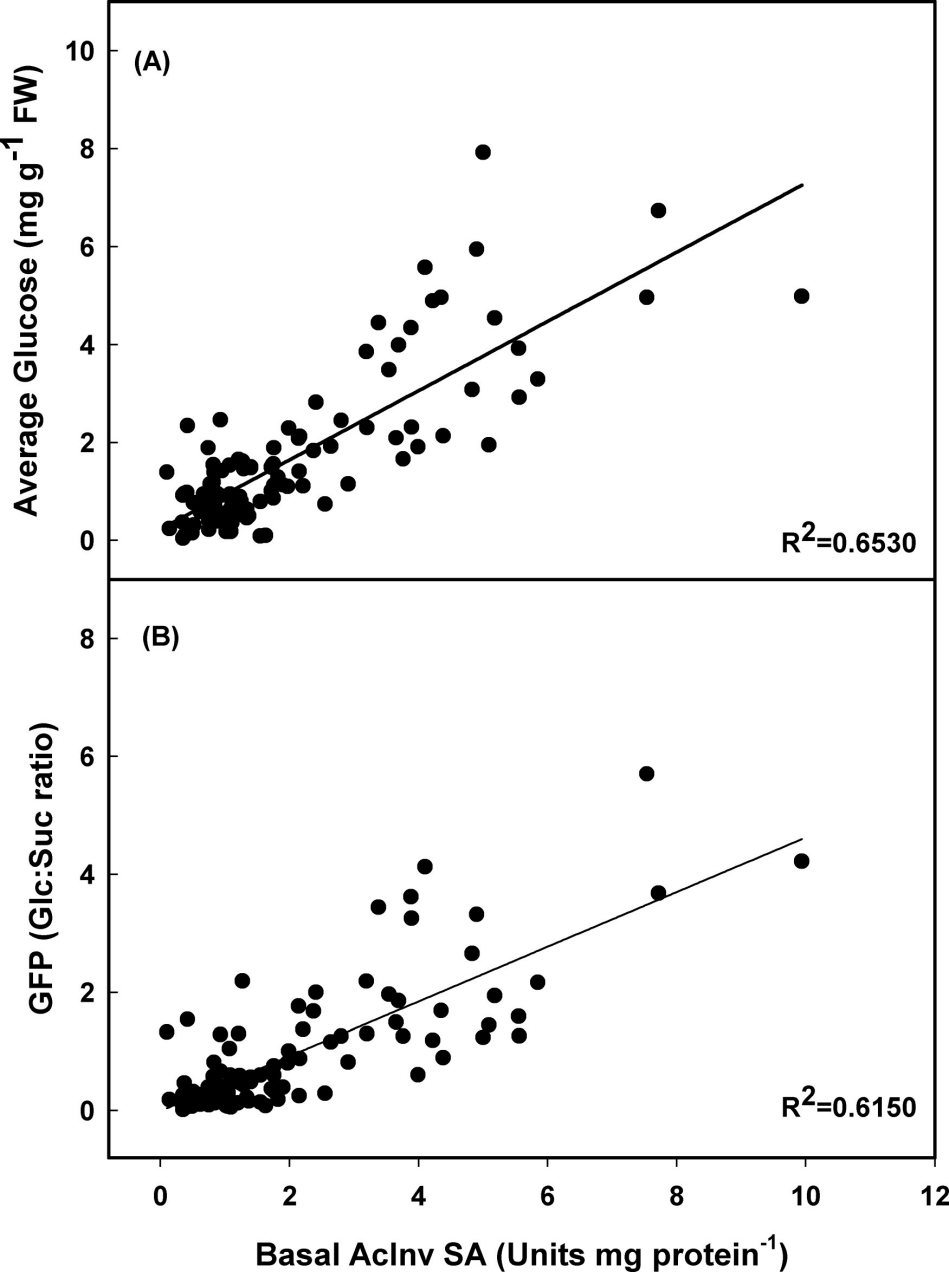
**Relationship between basal VAcInv enzyme activity and (A) glucose concentration and (B) GFP across all potato clones (n = 146).** Enzyme activity was expressed as μmols glc formed h^-1^. Glucose concentration was expressed as μmols.

The concentration of RS has been directly associated to chip or French fry color [10,21,51]. French fry color has been shown to be more closely related with glc concentration than with fru, suc, or total sugars [52]. Coleman [52] reported that chip color was correlated with tuber glc contents irrespective of the detection method, cultivar, or growing or storage condition conditions. Therefore, glc concentration has been used as the CIS indicator in this study. A clear association was observed between the basal VAcInv enzyme activity and glc concentration (Fig 1A). Clones with low basal VAcInv enzyme activity accumulated less RS. As shown in S3 Table, most of the CIS resistant clones are from class A.

The average glc concentration in class A clones was 0.73 mg g^-1^ FW (Table 2).

**Table 2:** ANOVA and mean comparison of basal and total VAcInv enzyme activity over four years. Values within a column that have a letter in common are not significantly different in pairwise comparisons (α = 0.05). Pairwise comparisons were only made for effects that were statistically significant in the ANOVA model (α = 0.05).

Glucose concentration	Year				
(mg·g^-1^ fresh weight)					
CIS class	2006	2007	2008	2009	Average
					across years
A (12 lines)	0.77	0.62	0.75	0.76	0.73 b
B (13 lines)	1.14	1.18	1.01	1.28	1.15 b
C (8 lines)	3.70	3.84	4.66	4.30	3.80 a
Average across CIS classes	1.87	1.72	1.87	2.12	-
Effect of CIS class (P-value)	**<0.0001**				
Effect of year (P-value)	0.5773				
Effect of CIS class * year (P-value)	0.8962				
Sucrose concentration	Year				
(mg·g^-1^ fresh weight)					
CIS class	2006	2007	2008	2009	Average
					across years
A (12 lines)	2.39	2.68	1.99	2.51	2.39
B (13 lines)	2.53	2.64	2.54	2.28	2.50
C (8 lines)	2.29	2.12	2.11	1.37	1.97
Average across CIS classes	2.40	2.48	2.21	2.05	-
Effect of CIS class (P-value)	0.2154				
Effect of year (P-value)	0.6106				
Effect of CIS class * year (P-value)	0.8324				

Tundra and Waneta accumulated low levels of glc. Atlantic showed maximum variability in terms of glc over years among class A clones. Lelah, even though characterized as class B based on basal VAcInv enzyme activity, accumulated the lowest level of glc during cold storage (S3 Table) because it has low total VAcInv activity.

The level of glc was found to be very stable across years (P = 0.5773), as was the level of suc (P = 0.6106).

In general, class B clones showed higher levels of average glc compared to class A clones (1.15 vs. 0.73 mg per g FW), but this difference was not statistically significant. Therefore, reducing sugar concentration after storage cannot be used to differentiate class A from class B. Class C clones and cultivars had a significantly higher average tuber glc concentration (3.80 mg per g FW) than those in class A or B. Norvelley had the lowest average glc concentration of 2.09 mg per g FW in class C, whereas the highest average glc concentration of 5.84 mg per g FW was observed in Red Pontiac (S3 Table).

For commercial potatoes stored at 9°C or higher, glc levels of less than 0.35 mg/g FW (0.035 percent) for chipping potatoes and less than 1.2 mg/g FW (0.12 percent) for French fries have been recommended [53]. In this study, all potatoes were subjected to excessive cold stress to increase genotypic variation in RS accumulation between classes. Normally, commercially stored potatoes for processing are stored at warmer temperatures. During storage at 7.2°C to 10°C, glc values would be lower than those seen in S3 Table.

A clear trend of high average glc concentration was observed in class A, B, and C after 6 months’ storage at 5.5°C storage (S3 Table). The glc levels at 6 months’ storage at 5.5°C can be explained by basal VAcInv enzyme activity, with an R^2^ value of 0.6530 (Fig 1A). Clones with high levels of basal VAcInv activity accumulated high levels of RS (S3 Table). Studies have shown that VAcInv activity increases during cold storage [20,22,46]. Clearly, class C clones did not maintain their CIS resistance after 6 months’ storage at 5.5°C storage. The increase in VAcInv activity during cold storage depends on genotype, storage temperature and level of inhibitor protein present.

Clones in classes A and B have higher suc concentrations than those in class C (S4 Table), because low levels of basal VAcInv enzyme activity will hydrolyze less suc into glc and fru [43–45,47,54]. However, suc concentration was not significantly related to CIS class (P = 0.2154).

Potatoes in the class A would have the greatest potential for processing successfully from long-term storage at 7.2°C, compared to class B clones (intermediate resistance, S3 Table). However, both class A and B produce light chip color and are acceptable for processing potatoes.

### Total sugar formation and glucose forming potential (GFP)

**Fig 2:**
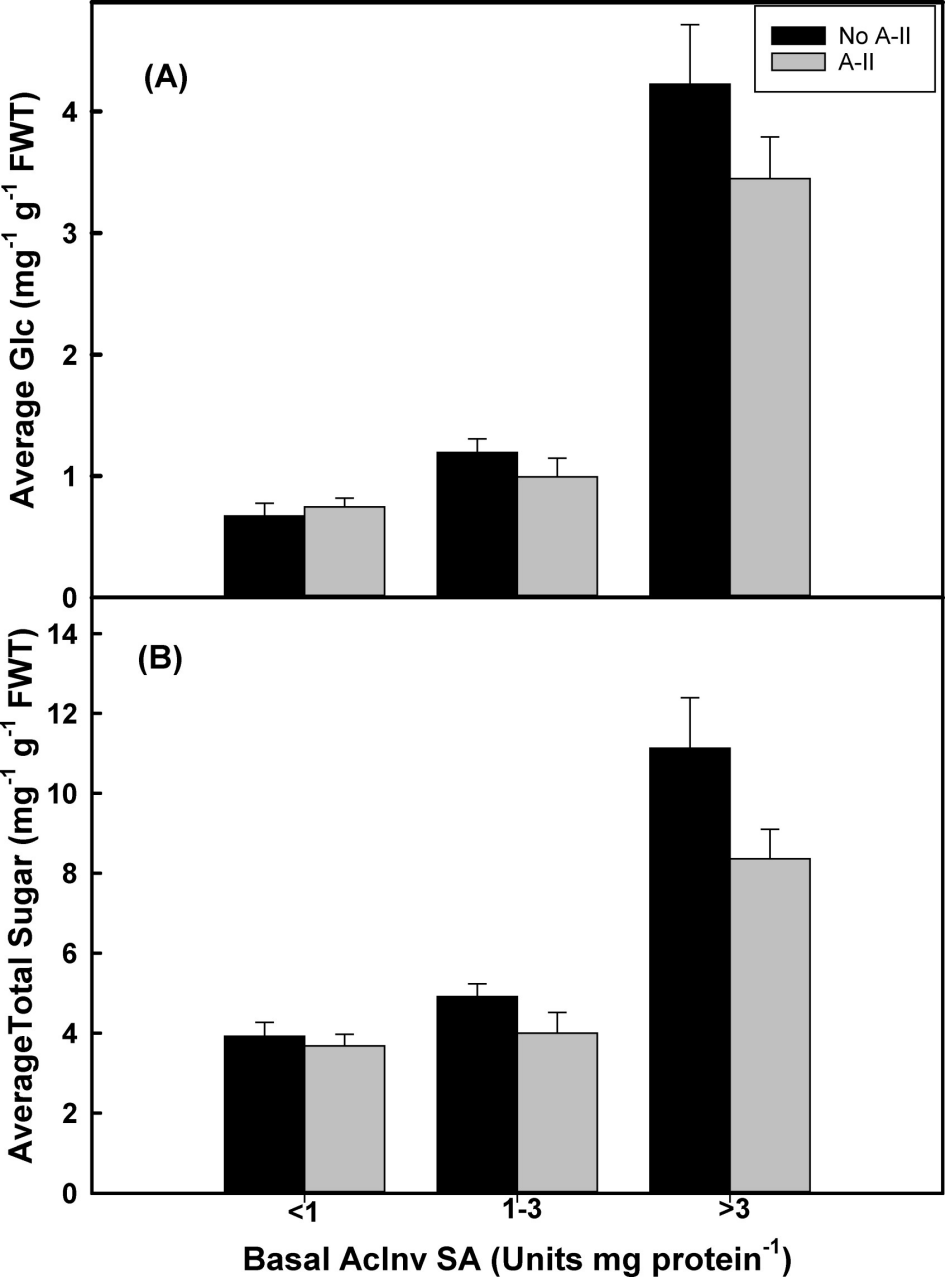
Average glc and total sugar concentration among all clones according to their assigned range of basal VAcInv enzyme activity units. All clones were stored for six months at 5.5°C. Reaction time was 1h at 37°C. One unit of activity is defined as the amount of extract necessary to catalyze the formation of 1μmols of glucose h^-1^. Data represents means of 3 reps ± SE.

Total sugar formation in a cultivar or clone could be a better reflection of the extent of starch conversion to sugars during cold storage and can be measured by sum up RS and suc. As shown in Fig 2A and 2B, clones and cultivars in class A and B did not show any substantial differences. However, class C had high levels of total sugar formation.

This could be attributed to high levels of starch hydrolyzing enzymes like starch phosphorylase and basal VAcInv enzyme activity, both are cold inducible [5–7,22,55]. As previously reported, the A-II isozyme of UGPase which limits suc formation [9,56], will not have a significant effect on RS accumulation when VAcInv is at high levels [10]. In this study, the presence of A-II isozymes of UGPase limits total sugar formation in all classes but the difference is clearly substantial in class C (Fig 2B). The A-II isozymes coupled with a low SA of VAcInv, have a synergistic effect in lowering glc concentrations among potato clones [9,10,56]. The catalytic properties of A-II isozymes of UGPase allows it to turn the Glc-1-P pool, towards glycolytic and other respiratory pathways [9,56].

Although total sugar formation could be a function of starch hydrolyzing enzymes and the enzymes in the hexogenesis pathway, for the CIS resistance, it is important to focus on the portion of suc hydrolyzed into RS. That is reflected by the glucose forming potential (GFP). In this study, a clear association was observed between basal VAcInv enzyme activity and GFP, R^2^ = 0.6150 (Fig 1B). The quantity of glc formed increased, along with its GFP. Similar trends were observed within the A+, B+, and C+ class clones. There was also a trend for less glc being formed, and a reduction in GFP, when A-II isozymes of UGPase were present compared to the minus classes (Fig 2B). Potato clones in the A class, regardless of the presence or absence of A-II’s, had the greatest resistance to sweetening at 5.5°C storage for six months. The GFP of the class C clones was 5-fold greater than classes A and B (Fig 2B). GFP (mg glc mg suc^-1^) quantifies the efficiency of VAcInv for converting the suc to glc. Soluble basal VAcInv enzyme activity has been shown to regulate the hexose-to-sucrose ratio in cold-stored potato tubers [19,47].

### CIS resistance prediction during cold storage

**Fig 3:**
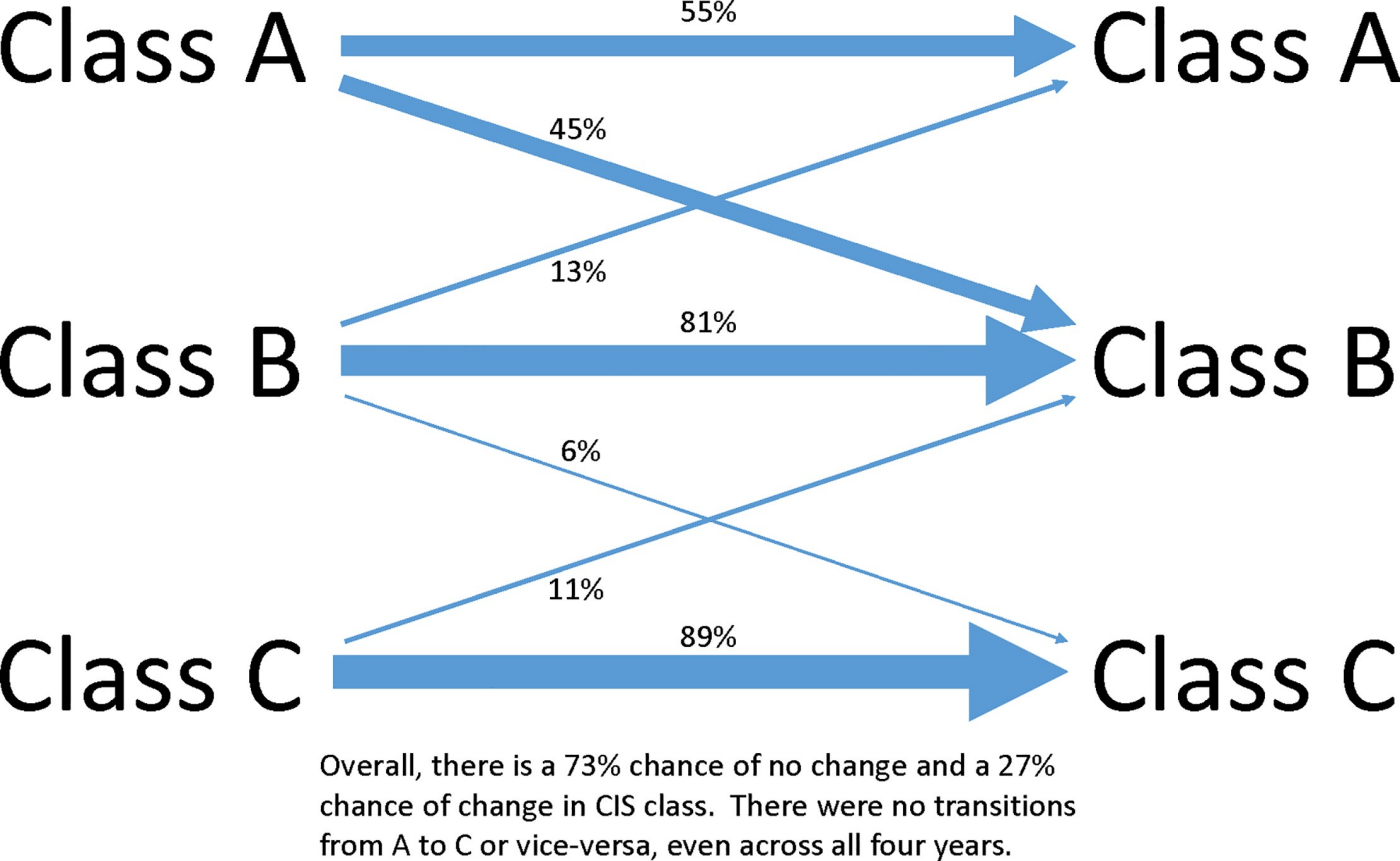
CIS resistance class transition over four years. All clones were evaluated for acid invertase enzyme activity, glucose concentration and sucrose concentration after six months storage at 5.5°C. They were assigned class A, class B or class C based on the acid invertase activity.

A clear relationship between basal VAcInv enzyme activity and RS level was found. Most of the clones and cultivars remained in their respective CIS resistance class over years. In the present study, 55% of the clones in class A stayed in class A, and the remaining clones switch to class B (Fig 3). This is probably due to the narrow range of basal acid invertase activity levels for class A.

Some clones characterized as class A shifted to class B and vice versa with glc level of below 1.2 mg per g FW.

Most clones in class B in one year remained in class B the next year, while a small percentage switched to class A (13%) or class C (6%) in the following year.

A clone in class C had an 89% chance of staying in class C and an 11% chance of switching to class B the following year.

There was a 73% chance of maintaining class from one year to the next, and a 27% chance of switching, overall. It is important to note that none of the class A clones shifted to class C or vice versa over four years.

Based on Friedman tests, clones did not have a consistent rank-order in CIS classes across years (P = 0.0447), suggesting that CIS category is not highly stable overall, consistent with the observed 27% overall probability of a clone changing classes between years (Fig 3). However, clones did maintain a consistent rank-order in basal VAcInv activity (P = 0.2263), indicating stability in this trait across years. The divergence in results for basal VAcInv activity compared to CIS class (which is entirely based on basal VAcInv activity) is likely due to the narrow range of VAcInv activity levels included in CIS class A (0–1 units/mg protein hour^-1^) and, to a smaller extent, class B (1–3 units/mg protein hour^-1^). 45% of clones in CIS class A and 19% of clones in class B in a given year changed classes in the following year, compared to 11% of clones in class C (Fig 3).

Previous studies have reported increases in VAcInv enzyme activity during cold storage, and the level of increase is the result of genotype and storage temperature interaction [34,35,57]. Over a four-year period, both of the biochemical markers described in this study remained stable (Fig 3).

Potato clones with up to 3 units of basal VAcInv activity remained acceptable in terms of processing quality. These biochemical markers can be used after harvesting to evaluate a tuber’s potential for accumulating sugars during storage. Tubers in class A can be stored for long term. Whereas, tubers in class B can be stored for mid-term. Tubers in class C at harvest can be eliminated.

DNA-based SNP markers associated with tuber glucose contents and fry color have been reported. Collectively these SNP markers explained between 24 to 46% of the variation in tuber sugar contents and fry color [33]. Compared to DNA-based markers, biochemical markers describe here 75% variation in tuber CIS resistance in this study.

As these biochemical markers were found stable over the years, they can be used to select parents for cross to achieve a high frequency of clone with better CIS resistance. Breeders will have the advantage of selecting parents of their choice with other desirable traits to get resulting progenies with a much higher frequency of clones with higher CIS resistance and other traits.

There are economic advantages for potato breeders in having a tool for the early selection of clones that have the potential for resisting sweetening in storage. Slater et al. in a review article described three main requirements for the adoption of MAS, which are consistent reproducibility, simple and straight-forwardness of the procedure and cost effectiveness. The marker system described here, provides such a tool. Considering the time for multiplication and storage of tubers and cost of DNA-based SNP markers, biochemical markers are very economical, effective and efficient. It is anticipated that VAcInv will be used as a guideline, coupled with the presence of A-II isozymes of UGPase, to help accelerate the selection of better potatoes with improved resistance to CIS in the future [4].

### Use of biochemical markers in potato breeding for CIS resistance

**Fig 4:**
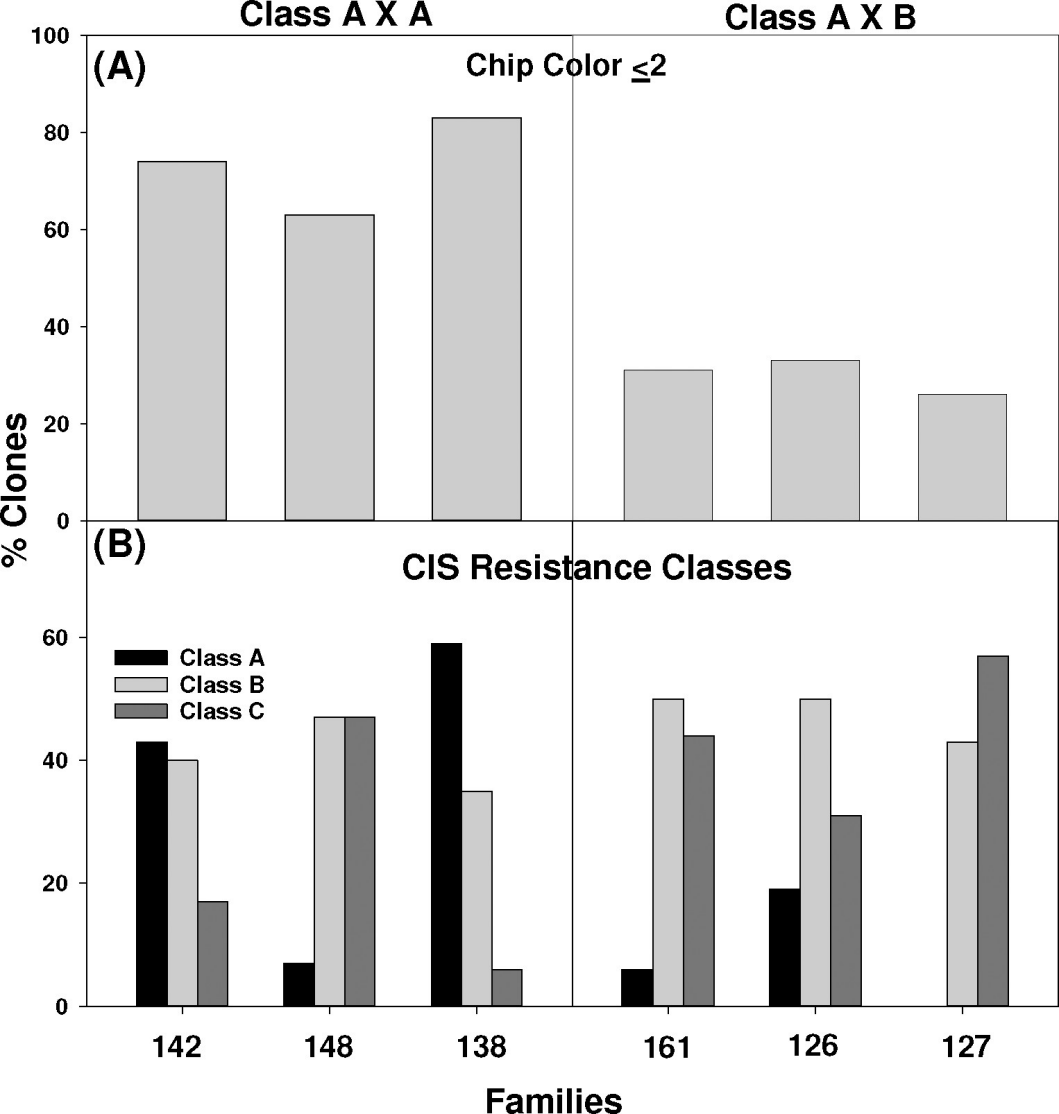
**Chip color (A) and CIS resistance classes (B) in six families generated based on the precise parent selection.** Parents and progenies from each family were analyzed for chip color (A) and CIS resistance class (B) after three months storage at 5.5°C.

The percentage of clones with chip color of ≤ 2 and RS of ≤ 1 mg/g fresh weight were significantly higher in families with both the parents from class A (Table 3 and Fig 4A, left panel) than in the families with only one parent from class A (Fig 4 A right panel).

**Table 3:** Cold-induced sweetening (CIS) resistance in various class combination families. Families highlighted in green are crosses between two parents from CIS class A. The remaining families are crosses between one class A parent and one class B parent.

					CC^2^	Glc^3^	CC^2^,	A-II	CIS Class^4^ (%),			AcInv Inhibitor^5^,	
Family	Female	Male	Class	n^1^	Harvest	Harvest	3 Months	Isozymes	3 Months @ 42°F			3 Months @ 42°F	
					% clones				A	B	C	Min	Max
142	MN99380-1Y	MN02696	A—* A-	35	97	97	74	0	43	40	17	0.654	13.837
148	ND860-2	MN99380-1Y	A+ * A-	16	73	73	63	50	7	47	47	0.610	15.602
138	MN02696	Waneta	A- * A+	19	100	69	83	32	59	35	6	0.605	25.317
161	W6609-3	Snowden	A- * B+	16	75	65	31	63	6	50	44	5.640	31.528
126	Atlantic	Waneta	B+ * A+	26	60	60	33	73	19	50	31	0.803	25.602
127	Atlantic	Lamoka	B+ * A+	23	52	52	26	78	0	43	57	2.708	23.157

**n^1^ Number of clones**

**CC^2^ Chip color 2/5 or less**

**Glc^3^ Glucose 1 mg·g^-1^ fresh weight or less**

**CIS Resistance Class^4^ A: < 1 total acid invertase unit; B: 1–3 units; C: > 3 units**

**AcInv Inhibitor^5^ Total acid invertase units**

Families 142, 148 and 138 had 73 to 100% of clones with chip color < 2, whereas families 161, 126 and 127, with one parent from class A and one parent from class B, had 52 to 75% of clones with chip color < 2 (Table 3). After 3 months’ storage at 5.5°C, the pattern remains similar, with slight decreases in total percentage. Here, it is important to note that both class A and class B produce acceptable chip color. In traditional potato breeding, parent selection for CIS resistance is based on chip color and reducing sugar levels after 6 months. Chip color or reducing sugar levels after storage cannot differentiate between class A and class B. Consequently, there will be a significant drop in the percentage of clones that are acceptable for processing. Data presented in Table 2 show that clones in class A and class B were not significantly different in terms of glc levels, but the difference in basal VAcInv activity was statistically significant (Table 1). This study clearly demonstrates a significant drop in selection efficiency just by changing only one parent from class A to class B.

In family 142 (MN99380-1Y X MN02696) represented by class A- X A-, none of the clones demonstrated presence of A-II isozymes of UGPase enzyme; however, family 148 represented by class A+ X A- had 50% clones with A-II isozymes of UGPase. Families with both parents having A-II isozymes (#126 and 127) had a higher percentage of clones with A-II isozymes of UGPase (Table 3).

Families (#142 and 138) had a higher percentage of clones with low levels of VAcInv enzyme activity and a higher percentage of clones in class A (Fig 4B, left panel). Family #148 which had both parents from class A, had a slightly different pattern (Fig 4B, left panel). Families with one parent from class A and one from class B had more clones with higher VAcInv activity and more clones in class C with no resistance to CIS than families 142 and 138(Fig 4B, right panel). High levels of VAcInv enzyme activity mask the effect of the A-II isozyme of UGPase.

The data clearly suggests that selection of parents from class A with A-II isozymes of UGPase and low inhibitor protein resulted in progeny with a much higher frequency of clones with improve CIS resistance, low RS and better French fry or chip color. Thus, by identifying better parents, cost should be reduced in subsequent evaluations due to a lower percentage of unacceptable clones.

These biochemical markers can be used effectively to screen a large number of early generation selections. Even though biochemical analysis is more cumbersome compared to simple chip color measurements, the time and cost involved in tuber increase and storage makes biochemical analysis an attractive alternative. In breeding programs, several thousand clones from various crosses are generated. It will take 1–2 years after single hill selection for tuber increase. Then, storing and chipping several thousand clones is labor intensive and costly. Most breeding programs select clones for a few years to reduce the number of clones before testing for CIS resistance. With the help of biochemical markers, promising clones with high CIS resistance can be selected much earlier in the selection process. Tubers after single hill selection can be cored with the help of a cork borer to obtain 5 grams of fresh tissue for biochemical analysis using the 96 well microplate procedure [34]. With the microplate based procedure a large number of samples could be analyzed in a short time, which will significantly reduce the number of clones to be planted and screened in subsequent years. One limitation with these markers is that they require mature tubers, in contrast to DNA markers where leaf tissue from green-house grown plantlets can be used. The biochemical analysis after 3 months storage at 5.5°C cold storage was a good indicator of CIS resistance in potato clones. Interestingly clones in families 142, 148 and 138 had low levels of acid invertase inhibitor protein as compared to clones in families 161, 126 and 127. This may be due to one parent in these families having low levels of acid invertase inhibitor protein.

VAcInv enzyme activity controls the glc:suc ratio [19] and is the result of the balance between the enzyme and inhibitor proteins [48]. The regulation of VAcInv activity by inhibitor protein is not well understood [58]. Studies have shown that VAcInv enzyme activity increases during long-term storage. The fold increase in VAcInv activity during cold storage is the result of the interaction among cultivar, storage temperature and the invertase inhibitor levels in the cell.

Analysis of preliminary data revealed the significance of acid invertase inhibitor protein. Potato clones with low levels of VAcInv and low invertase inhibitor protein had the best CIS resistance. Parents MN99380 and Waneta with low VAcInv and invertase inhibitor levels, had a higher percentage of clones with low reducing sugar levels.

A thorough understanding of VAcInv activity and its interaction with inhibitor proteins will enable us to better understand the accumulation of reducing sugar during long-term storage. Further investigations are required to understand the interaction of VAcInv and invertase inhibitor protein in regulating CIS resistance in cold-stored potato tubers.

These markers are available for use through University of Minnesota Office of Technology Commercialization.

http://license.umn.edu/technologies/20130267_assessing-cold-induced-sweetening-of-potato-varieties.

## Conclusion

As these biochemical markers were found stable over the years, it is suggested that two regulatory enzymes, UGPase and VAcInv, can be used as stable prognostic biochemical markers for CIS resistance for precise selection of parents to achieve a higher percentages of clones with improved CIS resistance and to screen large number of clones in a breeding program. Data presented clearly showed a significant drop in the percentage of clones with high CIS resistance by changing one parent from class A to class B. Differentiation of these two CIS resistance classes was not possible with traditional methods.

## Supporting information

S1 Table Basal acid Invertase enzyme activity after 6 months storage at 5.5°C over four years. (DOCX)

S2 Table Total acid invertase enzyme activity after six months storage at 5.5°C over four years. (DOCX)

S3 Table Glucose concentration after 6 months storage at 5.5°C over four years. (DOCX)

S4 Table Sucrose concentration after 6 months storage at 5.5°C over four years. (DOCX)

## Abbreviations

CIS: cold induced sweetening
DTT: dithiothreitol
Glc-1-P: Glucose-1-Phosphate
Glc-6-P: Glucose-6-Phosphate
GFP: glucose forming potential
HEPES: (N-[2-hydroxyl]piperazine-N’[ethanesulfonic acid])
PMSF: phenylmethylsulfonyl fluoride
PVDF: polyvinylidene fluoride
PVPP: polyvinylpolypyrrolidone
SPS: Suc-6-phosphate synthase
UGPase: UDP-Glc pyrophosphorylase
VAcInv: vacuolar acid invertase
SA: specific activity
Units: μmol glc·h^-1^
EA: Enzyme Activity, Units mg protein^-1^
FW: Fresh Weight
