# Supplementary material for: Identification and impact of stable prognostic biochemical markers for cold-induced sweetening resistance on selection efficiency in potato (Solanum tuberosum L.) breeding programs
Source: PLoS One. 2019 Dec 31;14(12):e0225411. doi: 10.1371/journal.pone.0225411 (PMC6938367; doi:10.1371/journal.pone.0225411)
Supplement: S1 Table — (DOCX) [file pone.0225411.s001.docx]

| Table 1. Basal acid Invertase enzyme activity after 6 months storage at 5.5C over four years. | | | | | | |
| --- | --- | --- | --- | --- | --- | --- |
| Clone | Basal Acid Invertase Activity (units/mg protein) | | | | | CIS Class |
|  | 2006-07 | 2007-08 | 2008-09 | 2009-10 | Average |  |
| ND5255-59 |  | 0.60 | 0.37 | 0.82 | 0.60 | A- |
| ND8304-2 |  | 0.52 | 0.10 | 1.20 | 0.61 | A- |
| ND8-14 | 0.77 | 0.38 | 0.82 | 0.67 | 0.66 | A- |
| Tundra |  | 0.78 | 0.49 | 0.72 | 0.66 | A- |
| Waneta |  | 0.34 | 1.03 | 1.02 | 0.80 | A+ |
| Dakota Pearl |  | 1.00 | 0.75 | 0.75 | 0.83 | A- |
| MSJ147-1 | 0.93 | 0.75 | 0.84 | 0.84 | 0.84 | A+ |
| Lamoka |  | 0.41 | 1.09 | 1.09 | 0.86 | A+ |
| MSN191-2Y |  | 0.14 | 1.55 | 1.05 | 0.91 | A+ |
| McBridge | 0.88 | 0.86 | 1.11 |  | 0.95 | A+ |
| Atlantic |  | 0.74 | 0.51 | 1.72 | 0.99 | A+ |
| ND5775-3 | 0.83 | 0.88 | 1.21 | 1.07 | 1.00 | A+ |
| Lelah | 0.35 | 1.55 | 1.63 |  | 1.18 | B+ |
| Sport 860 | 0.98 | 1.72 | 1.11 | 1.08 | 1.22 | B- |
| MSK061-4 | 1.04 | 1.20 | 1.34 | 1.37 | 1.24 | B- |
| Dakota Crisp | 1.97 | 1.40 | 0.76 |  | 1.38 | B+ |
| W2978-3 |  | 0.35 | 2.55 | 1.63 | 1.51 | B- |
| ND7192-1 |  | 2.15 | 0.62 | 1.82 | 1.53 | B- |
| Premier Russet | 1.75 | 1.90 | 0.95 |  | 1.53 | B- |
| W2683-2RUS | 0.93 | 1.34 | 2.37 |  | 1.55 | B- |
| A91814-5 | 1.76 | 1.75 | 1.29 |  | 1.60 | B+ |
| Clearwater Russet | 1.08 | 0.42 | 2.14 | 2.80 | 1.61 | B- |
| Ivory Crisp | 1.99 | 1.74 | 1.40 |  | 1.71 | B- |
| W2438-3Y | 2.64 | 1.76 | 1.25 | 1.27 | 1.73 | B- |
| Snowden | 1.23 | 2.91 | 1.97 | 2.21 | 2.08 | B+ |
| W2324-1 |  | 2.41 | 5.09 | 2.90 | 3.47 | C+ |
| Dark Red Norland | 3.38 | 3.54 | 3.88 |  | 3.60 | C- |
| NorValley | 3.2 | 3.76 | 3.89 |  | 3.62 | C+ |
| MN15620 | 5.55 | 3.99 | 2.16 | 5.56 | 4.32 | C- |
| Red Pontiac | 4.22 | 4.35 | 5.00 | 4.10 | 4.42 | C- |
| Shepody | 3.19 | 3.69 | 3.65 | 7.54 | 4.52 | C+ |
| Yukon Gold |  | 4.90 | 7.72 | 4.83 | 5.82 | C+ |
| Russet Burbank | 5.18 | 4.38 | 5.85 | 9.94 | 6.34 | C+ |
