# Supplementary material for: Identification and impact of stable prognostic biochemical markers for cold-induced sweetening resistance on selection efficiency in potato (Solanum tuberosum L.) breeding programs
Source: PLoS One. 2019 Dec 31;14(12):e0225411. doi: 10.1371/journal.pone.0225411 (PMC6938367; doi:10.1371/journal.pone.0225411)
Supplement: S2 Table — (DOCX) [file pone.0225411.s002.docx]

| Table 2. Total acid invertase enzyme activity after six months of storage, at 5.5°C over four years. | | | | | | |
| --- | --- | --- | --- | --- | --- | --- |
| Cultivars | Total Acid Invertase Activity (units/mg protein/hr) | | | | | CIS Class |
|  | 2006-07 | 2007-08 | 2008-09 | 2009-10 | Average |  |
| ND5255-59 | -- | 4.86 | 3.67 | 9.65 | 6.06 | A- |
| ND8304-2 | -- | 5.11 | 5.48 | 5.77 | 5.45 | A- |
| ND8-14 | 3.25 | 1.14 | 2.68 | 2.60 | 2.42 | A- |
| Tundra | -- | 0.60 | 2.45 | 3.59 | 2.21 | A- |
| Waneta | -- | 1.89 | 1.57 | 1.27 | 1.58 | A+ |
| Dakota Pearl | -- | 6.43 | 5.50 | 3.53 | 5.15 | A- |
| MSJ147-1 | 5.49 | 4.56 | 4.69 | 5.55 | 5.07 | A+ |
| Lamoka | -- | 0.80 | 3.71 | 1.64 | 2.05 | A+ |
| MSN191-2Y | -- | 3.99 | 2.98 | 3.01 | 3.33 | A+ |
| McBridge | 5.52 | 4.71 | 4.46 | -- | 4.90 | A+ |
| Atlantic | -- | 7.53 | 10.79 | 11.69 | 10.00 | A+ |
| ND5775-3 | 8.54 | 3.35 | 5.31 | 10.85 | 7.01 | A+ |
| Lelah | 1.62 | 1.74 | 4.43 | -- | 2.60 | B+ |
| Sport 860 | 14.02 | 12.71 | 14.46 | 3.11 | 11.08 | B- |
| MSK061-4 | 10.93 | 10.75 | 13.03 | 8.26 | 10.74 | B- |
| Dakota Crisp | 7.59 | 8.19 | 8.24 | -- | 8.01 | B+ |
| W2978-3 | -- | 4.53 | 4.23 | 4.42 | 4.39 | B- |
| ND7192-1 | -- | 4.36 | 12.23 | 6.13 | 7.57 | B- |
| Premier Russet | 29.42 | 11.59 | 17.26 | -- | 19.42 | B- |
| W2683-2RUS | 17.03 | 12.99 | 7.46 | -- | 12.49 | B- |
| A91814-5 | 23.16 | 11.65 | 23.39 | -- | 19.40 | B+ |
| Clearwater Russet | -- | 11.70 | 11.84 | 9.91 | 11.15 | B- |
| Ivory Crisp | 12.32 | 10.55 | 10.06 | -- | 10.98 | B- |
| W2438-3Y | 13.84 | 12.16 | 14.74 | 15.20 | 13.99 | B- |
| Snowden | 23.88 | 14.90 | 15.86 | 9.41 | 16.01 | B+ |
| W2324-1 | -- | 30.13 | 17.40 | 15.43 | 20.99 | C+ |
| Dark Red Norland | 30.71 | 21.91 | 25.86 | -- | 26.16 | C- |
| NorValley | 10.51 | 12.39 | 11.49 | -- | 11.46 | C+ |
| MN15620 | 13.43 | 18.15 | 16.68 | 13.20 | 15.37 | C- |
| Red Pontiac | 25.23 | 24.36 | 17.54 | 25.84 | 23.24 | C- |
| Shepody | 15.26 | 14.95 | 14.57 | 14.20 | 14.75 | C+ |
| Yukon Gold | -- | 12.40 | 9.78 | 10.17 | 10.78 | C+ |
| Russet Burbank | 42.09 | 37.43 | 27.28 | 26.61 | 33.35 | C+ |
