# Supplementary material for: Identification and impact of stable prognostic biochemical markers for cold-induced sweetening resistance on selection efficiency in potato (Solanum tuberosum L.) breeding programs
Source: PLoS One. 2019 Dec 31;14(12):e0225411. doi: 10.1371/journal.pone.0225411 (PMC6938367; doi:10.1371/journal.pone.0225411)
Supplement: S3 Table — (DOCX) [file pone.0225411.s003.docx]

| Table 3. Glucose concentration after 6 months storage at 5.5°C over four years. | | | | | | |
| --- | --- | --- | --- | --- | --- | --- |
| Clones | Glucose concentration (mg g^-1^ FWT) | | | | | CIS Class |
|  | 2006-07 | 2007-08 | 2008-09 | 2009-10 | Average |  |
| ND5255-59 | -- | 0.78 | 0.95 | 1.54 | 1.09 | A- |
| ND8304-2 | 0.58 | 0.31 | 1.39 | -- | 0.76 | A- |
| ND8-14 | 1.15 | 0.10 | 1.19 | 0.94 | 0.85 | A- |
| Tundra | 0.17 | 0.39 | 0.15 | -- | 0.24 | A- |
| Waneta | -- | 0.37 | 0.22 | 0.18 | 0.26 | A+ |
| Dakota Pearl | -- | 0.40 | 0.22 | 0.51 | 0.38 | A- |
| MSJ147-1 | 0.91 | 0.41 | 0.76 | 0.61 | 0.67 | A+ |
| Lamoka | -- | 0.98 | 0.18 | 0.18 | 0.45 | A+ |
| MSN191-2Y | -- | 0.24 | 0.79 | 0.37 | 0.47 | A+ |
| McBridge | 0.39 | 0.62 | 0.77 | -- | 0.59 | A+ |
| Atlantic | -- | 1.89 | 0.77 | 1.01 | 1.22 | A+ |
| ND5775-3 | 1.39 | 0.95 | 1.65 | 1.53 | 1.38 | A+ |
| Lelah | 0.04 | 0.09 | 0.10 | -- | 0.08 | B+ |
| Sport860 | 0.53 | 1.49 | 0.36 | 0.62 | 0.75 | B- |
| MSK061-4 | 0.33 | 0.52 | 0.46 | 0.50 | 0.45 | B- |
| DakotaCrisp | -- | 1.48 | 0.68 | 1.38 | 1.18 | B+ |
| W2978-3 | 0.34 | 0.92 | 0.74 | -- | 0.67 | B- |
| ND7192-1 | -- | 1.41 | 0.58 | 1.29 | 1.09 | B- |
| Premier Russet | 0.86 | 1.13 | 1.42 | -- | 1.14 | B- |
| W2683-2RUS | 2.46 | 0.63 | 1.83 | -- | 1.64 | B- |
| A91814-5 | 1.89 | 1.56 | 1.46 | -- | 1.64 | B+ |
| Clearwater Russet | 0.94 | 2.34 | 2.08 | 2.45 | 1.95 | B- |
| IvoryCrisp | 2.29 | 1.54 | 1.50 | -- | 1.78 | B- |
| W2438-3Y | 1.92 | 1.12 | 0.80 | 1.62 | 1.37 | B- |
| Snowden | 0.89 | 1.15 | 1.10 | 1.11 | 1.06 | B+ |
| W2324-1 | 1.94 | 2.82 | 1.95 | -- | 2.24 | C+ |
| Dark Red Norland | 4.44 | 3.48 | 4.34 | -- | 4.09 | C- |
| NorValley | 2.3 | 1.66 | 2.31 | -- | 2.09 | C+ |
| MN15620 | 3.92 | 1.91 | 2.12 | 2.92 | 2.72 | C- |
| Red Pontiac | 4.89 | 4.96 | 7.92 | 5.57 | 5.84 | C- |
| Shepody | 3.85 | 3.99 | 2.09 | 4.96 | 3.72 | C+ |
| Yukon Gold | -- | 5.94 | 6.73 | 3.08 | 5.25 | C+ |
| Russet Burbank | 4.54 | 2.13 | 3.29 | 4.98 | 3.74 | C+ |
