# Supplementary material for: Identification and impact of stable prognostic biochemical markers for cold-induced sweetening resistance on selection efficiency in potato (Solanum tuberosum L.) breeding programs
Source: PLoS One. 2019 Dec 31;14(12):e0225411. doi: 10.1371/journal.pone.0225411 (PMC6938367; doi:10.1371/journal.pone.0225411)
Supplement: S4 Table — (DOCX) [file pone.0225411.s004.docx]

| Table 4. Sucrose concentration after 6 months storage at 5.5°C over four years. | | | | | | |
| --- | --- | --- | --- | --- | --- | --- |
| Clone | Sucrose concentration (mg g^-1^ FWT) | | | | | CIS Class |
|  | 2006-07 | 2007-08 | 2008-09 | 2009-10 | Average |  |
| ND5255-59 |  | 2.90 | 2.05 | 2.69 | 2.55 | A- |
| ND8304-2 | 2.88 | 1.09 | 1.05 |  | 1.67 | A- |
| ND8-14 | 3.21 | 2.45 | 2.72 | 3.97 | 3.09 | A- |
| Tundra |  | 1.91 | 2.13 | 1.30 | 1.78 | A- |
| Waneta |  | 3.42 | 2.39 | 2.20 | 2.67 | A+ |
| Dakota Pearl |  | 3.27 | 2.34 | 1.41 | 2.34 | A- |
| MSJ147-1 | 1.37 | 2.14 | 1.65 | 4.85 | 2.50 | A+ |
| Lamoka |  | 4.36 | 2.92 | 3.25 | 3.51 | A+ |
| MSN191-2Y |  | 1.33 | 1.32 | 1.22 | 1.29 | A+ |
| Mcbridge | 2.78 | 2.11 | 1.62 |  | 2.17 | A+ |
| Atlantic |  | 4.83 | 2.44 | 2.76 | 3.34 | A+ |
| ND5775-3 | 1.71 | 2.38 | 1.27 | 1.46 | 1.71 | A+ |
| Lelah | 3.08 | 0.64 | 1.30 |  | 1.67 | B+ |
| Sport860 | 2.1 | 2.32 | 3.50 | 1.04 | 2.24 | B- |
| MSK061-4 | 4.69 | 4.11 | 2.08 | 3.22 | 3.53 | B- |
| DakotaCrisp | 2.52 | 2.64 | 2.44 |  | 2.53 | B+ |
| W2978-3 |  | 3.49 | 2.58 | 1.34 | 2.47 | B- |
| ND7192-1 |  | 5.71 | 5.71 | 6.86 | 6.09 | B- |
| Premier Russet | 2.38 | 2.88 | 3.55 |  | 2.94 | B- |
| W2683-2RUS | 1.92 | 1.17 | 1.09 |  | 1.39 | B- |
| A91814-5 | 3.16 | 4.65 | 3.39 |  | 3.73 | B+ |
| Clearwater Russet |  | 1.52 | 1.18 | 1.95 | 1.55 | B- |
| IvoryCrisp | 2.28 | 2.31 | 3.08 |  | 2.56 | B- |
| W2438-3Y | 1.66 | 1.49 | 1.74 | 0.74 | 1.41 | B- |
| Snowden | 1.51 | 1.41 | 1.37 | 0.81 | 1.28 | B+ |
| W2324-1 |  | 1.41 | 1.35 | 1.33 | 1.36 | C+ |
| Dark Red Norland | 1.29 | 1.77 | 1.20 |  | 1.42 | C- |
| NorValley | 1.77 | 1.32 | 0.71 |  | 1.27 | C+ |
| MN15620 | 2.46 | 3.17 | 2.42 | 2.32 | 2.59 | C- |
| Red Pontiac | 4.13 | 2.94 | 6.41 | 1.35 | 3.71 | C- |
| Shepody | 1.76 | 2.15 | 1.40 | 0.87 | 1.55 | C+ |
| Yukon Gold |  | 1.79 | 1.83 | 1.16 | 1.59 | C+ |
| Russet Burbank | 2.34 | 2.39 | 1.52 | 1.18 | 1.86 | C+ |
